# Human-like hopping in machines: Feedback- versus feed-forward-controlled motions

**DOI:** 10.1007/s00422-018-0788-4

**Published:** 2018-10-28

**Authors:** Jonathan Oehlke, Philipp Beckerle, André Seyfarth, Maziar A. Sharbafi

**Affiliations:** 1 Institut für Mechatronische Systeme im Maschinenbau, Technische Universität Darmstadt, Otto-Berndt-Straße 2, 64287 Darmstadt, Germany; 2 Elastic Lightweight Robotics Group, Robotics Research Institute, Technische Universität Dortmund, Otto-Hahn-Straße 8, 44221 Dortmund, Germany; 3 Lauflabor Locomotion Laboratory, Institute of Sport Science, TU Darmstadt, Magdalenenstr. 27, 64289 Darmstadt, Germany; 4 Control & Intelligent Processing Center of Excellence and the School of Electrical and Computer Engineering, College of Engineering, University of Tehran, 14395-515 Tehran, Iran

**Keywords:** Template-based control, Hopping with segmented leg, Virtual model control, Energy management

## Abstract

Template models of legged locomotion are powerful tools for gait analysis, but can also inspire robot design and control. In this paper, a spring-loaded inverted pendulum (SLIP) model is employed to control vertical hopping of a 2-segmented legged robot. Feed-forward and bio-inspired virtual model control using the SLIP model are compared. In the latter approach, the feedback control emulates a virtual spring between hip and foot. The results demonstrate similarity of human and robot hopping. Moreover, the feedback control proves to simplify and improve hopping control. It yields better perturbation recovery and locomotion adaptation and is even easier to tune. Thus, human-like hopping is achievable using a rather simple template-based controller, which ensures the required performance, robustness and versatility.

## Introduction

Reliable and robust control of legged locomotion under the influence of disturbances is indispensable to create legged robots that operate under real-life circumstances. Stabilization of robotic systems under the influence of different perturbations challenges classical control schemes (Siciliano and Khatib 2008). Feed-forward control methods (Vanderborght et al. 2011; Raibert and Brown 1984), negative feedback approaches (Zeglin and Brown 1998; Ahmadi and Buehler 1999) and their combinations (Haeufle et al. 2012) are widely spread for locomotion control. However, perturbations and environmental uncertainties make the situation more complex and difficult to control (Siciliano and Khatib 2008). When relying on pre-planned trajectories, different parameters need to be determined for each gait phase and situation (Siciliano and Khatib 2008). These task dependencies increase the difficulty of creating general control approaches. One option to cope with such challenges is using bio-inspired strategies. Biological legged structures, as robust and efficient locomotor systems, can be considered as templates for design and control, (Siciliano and Khatib 2008; Moro et al. 2013; Karakasiliotis et al. 2013).

**Fig. 1:**
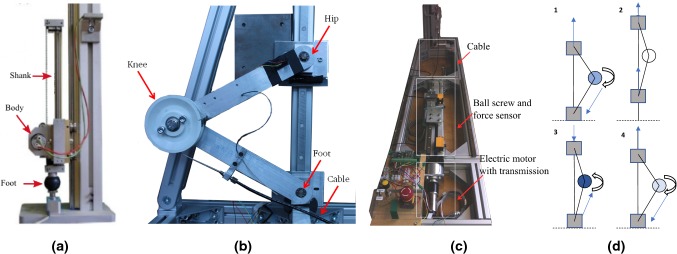
**a** MARCO-Hopper with prismatic leg (picture adopted from Kalveram et al. (2012)), **b** MARCO-Hopper II with a segmented leg and **c** the separate modular actuation mechanism, connected by a cable, **d** schematic of hopping motion, darker colors at knee mean higher actuation

Simple control, robust reaction to perturbations and high energy efficiency are the goals of a smart morphological design (Iida and Tedrake 2010). An example of a design that supports the desired movement is the passive dynamic walker, in which the robot can take steps without control (McGeer 1990). The combination of a self-stabilizing design and simple control strategies [termed as *exploitive control* in Kalveram and Seyfarth (2009)] can be used to create mechanisms for legged locomotion in the presence of rough terrain (Iida and Tedrake 2010). Through *exploitive control*, the controller benefits from the dynamics of the system instead of tracking a desired trajectory with the necessity of optimally tuned control parameters (Kalveram and Seyfarth 2009). This approach represents a rather descriptive concept, i.e., template (Full and Koditschek 1999), using abstract level of system dynamics. Hence, some physical constraints are not considered in contrast to classical control with detailed analytic models [i.e., Anchor Full and Koditschek (1999)]. For example in Kalveram et al. (2012), a hopping test bed called MARCO-Hopper was used to evaluate different energy management methods to realize stable hopping in the presence of losses and perturbations. Therefore, an extension of the spring-loaded inverted pendulum (SLIP) approach is applied to model human hopping (Blickhan 1989a). It has been shown that a constant supply of a certain amount of energy during each hopping cycle leads to terrain-following hopping, mimicking human hopping (Kalveram et al. 2012).

In this article, we extend the bio-inspired control of the MARCO-Hopper (Kalveram et al. 2012) to the MARCO-Hopper II robot with a segmented leg and knee extensor (Fig. 1). Here, the performance of different control strategies to realize hopping motions is shown.

Bouncing is one of the basic locomotor sub-functions [15]. Here, a bio-inspired template model is utilized to mimic human hopping. In this article, our previously implemented exploitive control on MARCO-Hopper II (Oehlke et al. 2016) is extended using virtual model control (VMC) (Pratt et al. 1997) and compared to the feed-forward control method. Section 2 shows the development of the control strategies and the hardware design. In Sect. 3, simulation and experimental results of the robot hopping are described, and compared to human hopping. Section 4 discusses and concludes the paper and presents potential future studies.

## Methods

In this study, a feed-forward controller and an exploitive approach based on a SLIP model are compared regarding their abilities in disturbance rejection in simulations and robotic experiments. To assess the control results, those are compared to human hopping experiments and key performance indicators, i.e., hopping height, frequency and duty factor, and robustness against perturbations, are investigated.

In contrast to the conservative SLIP model, which requires no compensation of energy losses, stable hopping without energy management is not achievable in a real robotic system (Kalveram et al. 2012). The energy management of the MARCO-Hopper robot (Fig. 1a) was developed based on the SLIP model (Kalveram et al. 2012). Yet, the segmented multi-body structure of the human leg is less similar to the SLIP model and the transferability of control strategies to such a mechanism remains as an open issue. To tackle this issue, the MARCO-Hopper II robot consisting of two links mimicking shank and thigh (with length $l_{\text {l}}$), which are connected by a knee joint, was developed, as shown in Fig. 1b. A mass ($m_1$) at the top point resembles the upper body, and the weight ratios between body, leg and foot are designed to approach those of humans. The knee is actuated via a cable (with stiffness $k_{\text {c}}$) attached to a pulley (with radius *r*) and exhibits an angular limitation of $\phi _\text {max}=120^\circ$ that prevents an over-extension of the leg and transfers kinetic energy from the hip to the leg.

The knee actuator comprises a geared DC motor ($P=200$ W, $i_{\mathrm{max}}=30$ A) and a ball screw, which are placed aside to reduce the inertia of the moving leg (Fig. 1c). The positions of the hip and the foot are calculated with the angular data of the thigh measured by an IMU (inertia measurement unit) and a potentiometer position sensor, respectively. The ground reaction force and the actuator force are measured with strain gauge sensors (See Table 1 for more details)

**Table 1:** Properties of the test bed and control parameters

Motor	Maxon EC-4pole, $P=200$ W
Transmission	Maxon GP 42 C, $i=91/6$
Ball screw	Item KGT VK14, $i=314$
Length segments leg	$l_{{l}}=0.25$ m
Mass at the hip, ”body-mass”	$m_1=1.3$ kg
Mass of a segment	$m_2=0.1$ kg
Mass of the foot	$m_3=0.3$ kg
Mass at the knee	$m_4=0.3$ kg
Stiffness of the cable	$k_{c}=556650$ N/m
Radius of the pulley	$r=0.034$ m
Mass *m* SLIP model	$m=1$ kg
Basic virtual stiffness $k_0$	$k_0=65.4$ N/m

### Control

Trajectory tracking is the core of different locomotion control strategies (Kalveram and Seyfarth 2009). Locomotion can be defined as a hybrid system comprised of different dynamics (e.g., stance and flight) switching to each other (Siciliano and Khatib 2008), which makes control more complex. Different phases of ground contact and flight alternate and perturbations through changing ground properties or external forces complicate a flawless motion. Feedback control systems, relying on predefined trajectories, operate at their limits when it comes to real-time locomotion with uncertain environmental conditions (Siciliano and Khatib 2008. Hence, simple robust methods using low sensory information and computational power are preferred for locomotion control.

An exploitive control based on the virtual model control (VMC) approach (Pratt et al. 1997) is presented to overcome the aforementioned limitations as suggested in Oehlke et al. (2016). This method takes the system dynamical properties into account and uses minimal sensory information for control. Therefore, modeling, system identification, control of the simulated model and implementing on the hardware are the required steps. The proposed controller is inspired from the human reflex system, in which the controller is reactive to body parameter changes measured by a sensory system, e.g., muscle length or force used for proprioceptive feedback (Haeufle et al. 2012). In addition, a simple feed-forward approach is developed using a sinusoidal current pattern for the knee extensor. Applied to the system, a periodic motion of the leg is targeted. The control does not need sensor information and also does not have high computational costs. It has been shown that sinusoidal feed-forward approaches are able to produce stable locomotion [see, for example, Seyfarth et al. (2009) or Iida and Tedrake (2010)]. A second reason for working with a feed-forward approach is the similarity to central pattern generators (CPG) which are a biologically plausible way to generate periodic movements (Kalveram 1991; Haeufle et al. 2012) (here: sinusoidal patterns driving the actuator).

#### Feed-forward control concept

Feed-forward control approaches require deep system insight and precisely identified dynamics and loss parameters. Without proper adjustment, it is not possible to react to changing conditions due to the lack of feedback. Nevertheless, in this method tuning parameters is straightforward using small sensory information that simplifies implementation both in simulation and on the test bed. Therefore, it is an easy way to produce a hopping motion with MARCO-Hopper II. In this method, the desired current *i*(*t*) is given by

1 $$\begin{aligned} i(t)=i_{\text {max}}\sin (\omega _0 t+\phi _\text {c}) \quad -\,3\,\text {A} \le i(t) \le 30\,\text {A} \end{aligned}$$

in which $i_{\text {max}}$, $\omega _0$ and $\phi _\text {c}$ are the amplitude, frequency and phase shift of the desired oscillatory current, respectively. Limitations of the current are given by the maximal motor current of $i=30 \text { A}$. A current of $i=-3 \text { A}$ is necessary to drive back the carriage to the origin point, while the leg touches the ground after the hop and goes through the compression phase. The internal current controller of the drive train is programmed to track this desired function.

The system complexities as a multi-body structure, with unknown, position-dependent losses and non-ideal drive train properties, make it difficult to predict the resulting behavior. In order to make the two approaches comparable, the amplitude and frequency values of the feed-forward approach are chosen to resemble those observed using the VMC approach.

#### virtual model control as an exploitive control

**Fig. 2:**
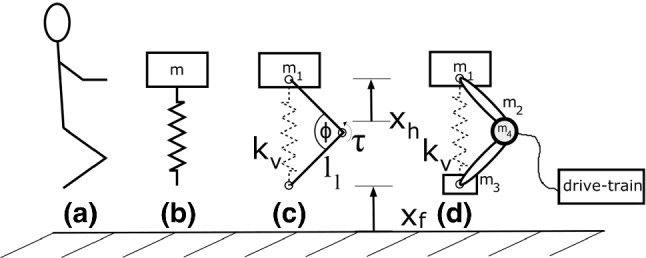
Evolution of models from human hopping to MARCO-Hopper II. **a** Human vertical hopping can be described by **b** the SLIP model, **c** a segmented leg mechanism with one (body) mass at the hip. The knee torque $\tau$ mimics the leg force represented by the virtual leg spring (with stiffness $k_{\text {v}}$). **d** Complete model of MARCO-Hopper II with distributed masses, energy dissipation effects and drive train

Human motions (Fig. 2a) can be modeled using various approaches: the template SLIP model (Fig. 2b), a segmented leg carrying a mass (Fig. 2c), or a complete multi-body simulation model of MARCO-Hopper II (Fig. 2d). While the complete model can represent details such as the actuator and transmission dynamics and losses, e.g., in the drive train or bearings, VMC is expected to mimic human hopping even when using the very reduced SLIP model. By implementing the controller on the MARCO-Hopper II test bed, the approach is evaluated experimentally on a two-segmented robotic mechanism.

With the virtual model control (VMC) approach (Pratt et al. 1997), the effects of a spring with the stiffness $k_{\text {v}}$ between hip and foot are mimicked, as shown in Fig. 2c. The torque $\tau$ at the knee is controlled in a manner to have the same effect on the leg as a spring would have. Obviously, vertical force cannot be generated in the fully stretched leg configuration because of singularity. This situation is avoided in human knee joint and also in the MARCO-Hopper II using mechanical constraints (locking mechanism in the robot). By mathematical manipulations, the equation of motion for the top mass *m* is obtained as

2 $$\begin{aligned} m\ddot{x}=\frac{\tau }{2\cos \left( \frac{\phi }{2}\right) l_\text {l}}-m g. \end{aligned}$$

Here, one goal is to resemble the behavior of a spring–mass system with a spring length $l_0$ and a spring constant $k_v$. Therefore, the driving terms of the spring–mass system and the multi-body system are set to be equal. Thus, if the knee torque $\tau$ is applied as follows, the structure moves like a spring–mass system.

3 $$\begin{aligned} \tau =2\cos \left( \frac{\phi }{2}\right) l_\text {l} k_{\text {v}} (l_0-x_\text {h}+x_\text {f}) \end{aligned}$$

in which $\phi$, $l_0$, $x_{\text {h}}$ and $x_{\text {f}}$ are the knee angle, the virtual leg spring rest length, the positions of the hip and foot, respectively. This approach is valid for a mass-less segmented leg with one mass at the hip *m*, shown in Fig. 2c. Although this relation for the complete model of MARCO-Hopper II including distributed masses in the legs (shown in Fig. 2d) is more complex, the same principle will be held. A system with distributed masses $m_1$, $m_2$ and $m_3$ for body, leg segments and the foot, respectively, mimics the behavior of a single mass oscillator *m* (see Fig. 2), if the torque $\tau$ is generated with the following law:

4 $$\begin{aligned} \tau = 2\cos \left( \frac{\phi }{2}\right) l_{l}&\left( \frac{k_{v} (l_0-x_h+x_f) -mg}{m} M_1 + M_2g\right) \nonumber \\ \text { with:}\nonumber \\ M_1=m_1&+ \frac{3}{4} m_2+ \frac{1}{4} m_4\nonumber \\ M_2=m_1&+m_2+\frac{m_4}{2} \end{aligned}$$

The desired hopping condition, e.g., the hopping height can be determined by tuning the virtual spring parameters: the rest length $l_0$ and the stiffness $k_{\text {v}}$. In all simulations and experiments, the initial condition is assumed to be with bent knee/compressed spring (maximum compression, *MC*). The hip and foot initial heights are set to $x_\text {h}=x_0$ and $x_\text {f}=0$, respectively. The virtual leg spring rest length is adjusted to the maximum leg length $l_0=0.5$ m of MARCO-Hopper II. The rest length of the spring $l_0$ is assumed to be constant and, thus, the hopping height can be adjusted by $k_\text {v}$. A more extended approach could modify $k_\text {v}$ and $l_0$ to change hopping conditions, e.g., hopping frequency and height.

In the SLIP model, the hopping frequency is determined by $f=\frac{1}{2 \pi }\sqrt{k/m}$, which is the natural frequency of the system. Considering the energy balance at MC and takeoff, the required condition for leaving the ground will be

5 $$\begin{aligned} k_{v}>\frac{2mg}{l_0-x_0};\quad k_0=\frac{2mg}{l_0-x_0}. \end{aligned}$$

A spring rate of $k_0$ would result in a periodic oscillation with a maximal deflection till the rest length of the spring. Satisfying the condition $k_v>k_0$ along with Eqs. (3) or (4), results in control of a desired hopping motion in the SLIP model and more complex multi-body models.

**Fig. 3:**
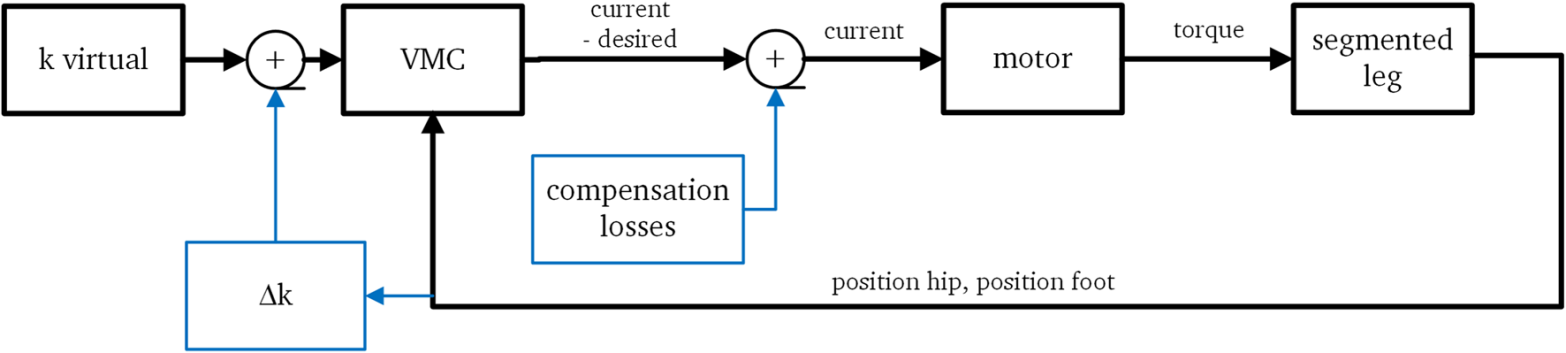
Control block diagram of MARCO-Hopper II. Black parts (lines and boxes) show the basic control mechanism for SLIP-based virtual model control (VMC). The blue parts include additional alternatives for energy management. Calculations of the desired force and a simplified drive train model are contained in the VMC block giving the desired motor current, all performed in real time (color figure online)

A block diagram of the control approach is presented in Fig. 3.

The control concept is shown with black lines, in which the VMC uses the distance between hip and foot, the actual length of the virtual spring, and the virtual stiffness to find the desired knee torque. To set the desired torque, the corresponding motor current is calculated using a motor model. In addition to this basic strategy, that relies on feeding back the actual leg length, the extended approach which is shown by the blue lines in Fig. 3 helps overcome energy losses. One approach could be injecting the computed amount of lost energy. Since the model is complex and uncertain, calculating the specific amount of lost energy is not straight forward. It was shown that for SLIP-based control of the MARCO-Hopper, injecting a constant amount of energy in each bounce results in stable hopping (Kalveram et al. 2012).

In this paper, changing the virtual spring stiffness during the movement by adding an additional stiffness term ${\varDelta }k$ is chosen to inject energy. Injecting a fixed amount of energy ${\varDelta }W$ greater than a threshold ${\varDelta }_T$ results in converging to a periodic stable vertical hopping . ${\varDelta }_T$ is equal to the losses during a hopping cycle. Because the prediction of the losses is challenged by uncertainties, the additionally injected amount of energy is adjusted during experiments to achieve a desired hopping motion. The spring stiffness adjustment starts with $k_v=k_0$ at MC and increases by adding ${\varDelta }k$ (Fig. 3).

6 $$\begin{aligned} \begin{aligned} k_{\text {v}}&=k_0+{\varDelta }k\\ \text {with: }{\varDelta }k&=\frac{6\text { }{\varDelta }W}{(l_0-x_0)^3} ((x_{\text {h}}-x_{\text {f}})-x_0)\\ \end{aligned} \end{aligned}$$

where $k_0$ serves as a hopping condition extracted from the SLIP model while ${\varDelta }k$ compensates losses and unmodeled effects. In Kalveram et al. (2012) it was shown that with this formulation the energy ${\varDelta }W$ is injected in the system during each hopping cycle in a biologically reasonable way, while moving up.

The virtual stiffness is set to zero during the downward motion and upward motion when the hip is above a threshold $x_+$. This means that the knee torque is set to zero and the leg leaves the ground because of the body inertial force. In downward movement, the virtual spring stiffness switches back to $k_v$ when the hip point reaches the point, $x_-$ which is above MC. This control method is selected due to sensory limitations of the test bed. The derivative of the measured hip position is used to detect motion direction.

7 $$\begin{aligned} k_{\text {v}}= & {} k_0+{\varDelta }k \quad \text {if}\;\;\; (x_\text {h}<x_-) \vee ([x_\text {h}<x_+] \wedge [\dot{x}_\text {h}>0])\nonumber \\ k_\text {v}= & {} 0 \quad \text {if}\;\;\; (x_\text {h}>x_+) \vee ([x_\text {h}<x_+] \wedge [\dot{x}_\text {h}<0]) \end{aligned}$$

The presented logic for changing the virtual spring stiffness is a reflex approach (Pavlov 2010). In this study, the leg length is considered as the main reflex signal to tune the virtual spring stiffness. The injection of a fixed amount of energy ${\varDelta }W$, using the stiffness increment by ${\varDelta }k$, determines the hopping height. Again the current of the motor is tuned to $i=-3$ A during the fall of the leg, to drive back the carriage holding the cable (“compensation losses” in Fig. 3).

### Experiment

#### Procedure of examinations

Simulations and experimental investigations of hopping in place including ground level perturbations are considered to compare the VMC and feed-forward control approaches. When humans confront with small changes of ground level, it is often not even noticed and the stability of the motion is not affected (Kalveram et al. 2012). Furthermore, adjustment of the hopping height to new desired values is investigated to evaluate control performance.

**Fig. 4:**
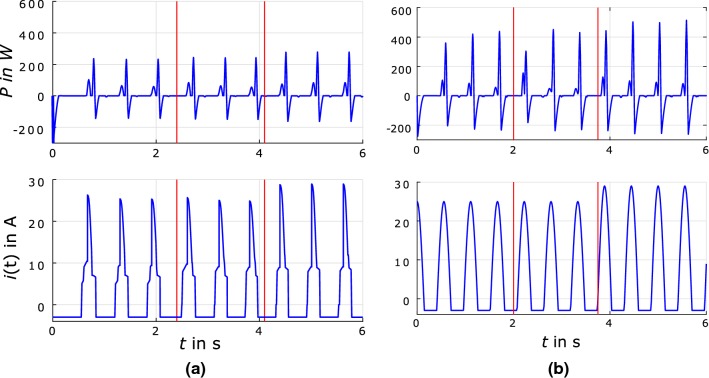
The power consumption *P* and the current pattern *i*(*t*) in MARCO-Hopper II simulations. Ground level change and hopping height increase are depicted by the red lines. (Left) The VMC approach with ${\varDelta }W=8\,\hbox {J}$ and ${\varDelta }W=10\,\hbox {J}$ after increase of hopping height and $x_-=0.35\,\hbox {m}$, $x_+=0.44\,\hbox {m}$ as reflex parameters. (Right) The feed-forward (FF) control with sinusoidal current pattern with $i_{\text {max}}=25\,\hbox {A}$ and $i_{\text {max}}=29\,\hbox {A}$ after increase of hopping height and a frequency of $f_0=1.8\,\hbox {Hz}$ with a phase lag of $\phi _\text {c}=\pi /2$ (color figure online)

**Fig. 5:**
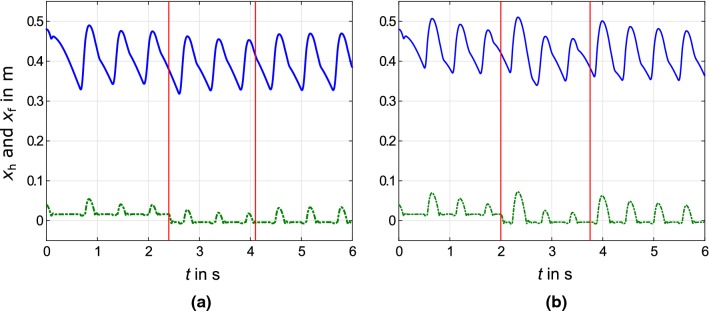
Simulation of MARCO-Hopper II with predicted hip $x_\text {h}$ and foot $x_\text {f}$ positions, shown by solid blue and dashed green lines, respectively. After the third hop, the ground level is decreased by ${\varDelta }x=0.02\,{\hbox {m}}$ and after six hops the desired hopping height increases (shown by red lines. **a** VMC approach with ${\varDelta }W=8\,\hbox {J}$ and ${\varDelta }W=10\,{\hbox {J}}$ after increase of hopping height and $x_-=0.35\,{\hbox {m}}$, $x_+=0.44\,{\hbox {m}}$ as reflex parameters. **b** Sinusoidal current pattern with $i_{\text {max}}=25{\hbox {A}}$ and $i_{\text {max}}=29{\hbox {A}}$ after increase of hopping height and a frequency of $f_0=1.8\,{\hbox {Hz}}$ with a phase lag of $\phi _\text {c}=\pi /2$ (color figure online)

*Simulations* First, the perturbation rejection is examined by downward movement of the ground level (${\varDelta }x=-0.02\,\hbox {m}$) The second task is to change the desired hopping height during a constant hopping motion. In the case of the feed-forward approach, the amplitude of the sinusoidal pattern $i_\text {max}$ is raised (see Sect. 2.1.1). For changing the hopping height with the exploitive control approach, the value for ${\varDelta }W$ as the injected energy is raised. Both tests are combined in one simulation that starts in the flight phase of hopping where the foot has an initial position of $x_{\text {f}}=0.02$ m. After the third hop, the ground level is changed, and three hops later the desired hopping height is raised by an adaptation of the control parameters (see red vertical lines in Figs. 4, 5).

*Experiments* In a set of experiments, the performance of the control methods is investigated on the test bed and compared to human hopping. Experiments start in the flight phase of the hopping motion, with $x_{\text {f,0}}=0.02$ m and $x_{\text {h,0}}=0.46$ m, and the ground level is changed by ${\varDelta }x=-0.02\,\hbox {m}$ during flight phase. Measurement of the hip position $x_\text {h}$, the foot position $x_\text {f}$ and ground reaction forces $F_\text {GRF}$ as well as the real-time control are performed with the frequency $f_{\text {M}}$. The ground reaction force is normalized with respect to the weight of the segmented leg ($F_{\text {m,tot}}=14.715$ N). Positions of the hip and the foot are normalized with the maximum leg length of the robotic leg ($l_{\text {tot}}=0.44$ m). The mechanical power *P* is measured at the carriage holding the cable. A force sensor is included in the test bed to experimentally determine the power and compare it to the simulation results.

We have also performed experiments with increasing hopping height. The results were qualitatively similar to downward perturbations, but because of limitations of the test bed it was difficult to perform exact repeatable perturbations. In addition, for comparison to the previous work of Kalveram et al. (2012), which considered ground level decreases only, we focused on this kind of perturbations.

*Experimental criteria* The experimental data are analyzed regarding the mean absolute and relative hopping height (to the leg length) as well as the mean hopping frequency over all performed hops. These values are used to compare the resulting motions with the human experiment. Further, the relation between ground reaction force and leg length is analyzed. This work-loop method which was developed for the examination of muscle properties in Josephson (1985) is also used to analyze human gaits (Maykranz et al. 2013). In the context of locomotion, it describes the relationship between leg length and ground reaction force during periodic motions, e.g, walking/running.

In addition, efficiency is examined in terms of energy consumption. Therefore, the ratio between the change of potential energy during flight phase $E_\text {pot}$ and the injected energy is calculated. As a simplification, $E_\text {pot}$ is assumed to be equal to the energy that is necessary to lift the system mass to the hopping height $\hat{x}_\text {f}$.

8 $$\begin{aligned} E_\text {pot}= (m_1 + 2\;m_2 + m_3 + m_4)\;g\;\hat{x}_\text {f} \end{aligned}$$

This conservative estimation ensures to reach the desired or higher hopping heights. To determine the injected energy, the measured force at the cable is used. The integral of the measured power *P* over a certain time interval is equal to the energy consumption *E* during this interval. When the leg begins to apply force to the ground $F_\text {GRF}>F_\text {W}$ and when the foot leaves the ground $F_\text {GRF}=0$ are chosen as two interval limits. The power applied to the system during this period results in a certain hopping height.

9 $$\begin{aligned} E=\sum {(P\;{\varDelta }t)} \end{aligned}$$

The relationship between potential and total energy is used to assess the efficiency $\eta _\text {h}$ of a single hop:

10 $$\begin{aligned} \eta _\text {h}=\frac{E_\text {pot}}{E} \end{aligned}$$

### Simulation model

The aforementioned models (Fig. 2) are simulated in Matlab Simulink and SimMechanics with the ODE4 (Runge-Kutta) solver and a fixed step size of 0.2 ms. To account for the rather complex friction effects in the drive train and the Bowden cable, friction forces $F_\text {fr}$ were modeled based on an adapted *Stribeck*-model (Krämer and Kempkes 2014).

11 $$\begin{aligned} \begin{aligned} F_{\text {fr}}&=F_{\text {St}}\text { }\tanh (800\;\dot{x}) + (F_{\text {v}} \dot{x}) \\ F_{\text {St}}&=F_{\text {c}}+(F_{\text {s}}-F_{\text {c}}) \exp \left( -\left| \frac{\dot{x}}{v_{\text {s}}}\right| ^{\delta _{\text {s}}}\right) \\ \end{aligned} \end{aligned}$$

$F_\text {v}$, $F_\text {c}$ and $F_\text {s}$ describe the force components of the velocity dependent, coulomb friction and stiction terms, respectively. $v_\text {s}$ presents the actual velocity of the moved parts, while $\delta _\text {s}$ describes the shape of the exponential function. Each value is fitted to the behavior on the test bed. The ground impact model based on a nonlinear spring–damper model is adopted from Vu et al. (2015).

The complete parametrization and the developed models are available in Oehlke (2015).

## Results

### Simulation studies

In order to compare the two control approaches, the parameters are selected to achieve the most similar performance. In VMC method, if the injected energy ${\varDelta }W$ is higher than the losses in the system, a stable hopping motion with a certain hopping height is achieved. For raising the hopping height, the value of ${\varDelta }W$ needs to be increased. In the simulations, this injected energy has been adjusted to ${\varDelta }W=8\,\hbox {J}$ keeping the constraints on the motor current. We use the VMC current patterns (Fig. 4 (left, bottom)) to characterize feed-forward control.

The frequency of the current oscillation comes close to $f=1.8\,\hbox {Hz}$, while the maximum current is about $i_\text {max}=25\,\hbox {A}$. These similar values besides phase lag $\phi _\text {c}=\pi /2$ (see Sec. 2.1.1) are selected for the sinusoidal current pattern in feed-forward approach, shown in Fig. 4 (right, bottom). These comparable current patterns result in similar periodic movements, shown in Fig. 5 by the hip $x_\text {h}$ and the foot $x_\text {f}$ positions.

Table 2 presents the average values and standard deviations of hopping height and the ground reaction force of ten consecutive undisturbed hops (20 s, no change of ground level, no change of ${\varDelta }W=8\hbox {J}$ or $i_\text {max}=25\hbox {A}$). Taking the measurement resolution into account, hopping height does not vary when applying the VMC while the feed-forward approach leads to considerable standard deviations. Yet, the mean hopping height achieved with the VMC is below the hopping heights of the FF approach. Calculating the average hopping frequency across the whole duration of the experiment except the first hop yields $f_\text {VMC}=1.7\,\hbox {Hz}$ for the VMC approach and $f_\text {FF}=1.8\,\hbox {Hz}$ for the feed-forward.

The first vertical line shows the occurrence of the downward *ground level perturbation*. In response, both control methods generate one hop with an increased height following the perturbation. For the VMC approach, this change is distinctly smaller than for the feed-forward one. After the perturbation, a convergence to the nominal hopping is observed. To define a nominal hopping pattern, the behavior in the absence of disturbances is recorded.

**Table 2:** Means *m* and standard deviations $\sigma$ of hopping height and ground reaction force over $T=20\,\hbox {s}$ of undisturbed hopping motions with virtual model control and feed-forward control

	VMC		FF	
	*m*	$\sigma$	*m*	$\sigma$
$\hat{x}_\text {f}$ (cm)	1.83	0.02	3.29	1.1
$F_\text {GRF}$ (N)	75.02	0.98	100.35	12.72

*Hopping height adjustment* points out the control performance through tracking new desired commands. For raising the hopping height during the motion, the injected energy is set to ${\varDelta }W=10\,\hbox {J}$. This is equivalent to a current raise to $i_\text {max}=29\,\hbox {A}$. The resulting motion and current patterns are shown in Figs. 4 and 5, after the second red line. The VMC approach results in an immediate change of hopping height, with no visible transient oscillation. With the feed-forward approach, it takes multiple hops to reach a constant hopping height. Similar to the findings in Haeufle et al. (2012), the feed-forward control can stabilize the movement and compensate for perturbations but requires long settling times with higher variations. In VMC, additional feedback to the controller results in more responsive (close to deadbeat) behavior. Additionally, this shows that the VMC approach yields similar energy management as in humans and in the MARCO-Hopper (Kalveram et al. 2012).

The current pattern (shown in Fig. 4) is the desired current, resulting from the control scheme. In the simulation, the motor is driven exactly with this current while there is a deviation in the current during experiments, resulting from current control dynamics. Comparing the control methods in Fig. 4 shows that the feed-forward approach needs a noticeablly higher power level, especially at impacts (about twice compared to the VMC approach). These peaks that go over $P=400\,{\hbox {W}}$ are mechanical impacts, happening when the leg hits the knee stop. With such a higher peak power, the motor comes to a sudden forced stop, which strains the cable and the drive train and leads to high force values at the force sensor. Additionally, the considerably higher hopping is resulted from the higher energy injection.

The region around the first peak of the power course for each hop shows the power that leads to an acceleration of the motion. The power is consumed by structure acceleration and the overcoming of internal resistances. This first peak happens in a very early stage of the leg extension. Another difference between the power curves is the level of consumed power just before the full extension of the leg (the moment before the second, higher power peaks. With the VMC approach, the power goes always back to zero ($x_\text {h}>x_+$), while the feed-forward approach still has a positive amplitude before reaching full extension. Additionally, in both methods, the peak current lies in the first half of leg extension. This peak happens simultaneously to the first peak of power consumption. In contrast to feed-forward method in VMC, the current goes back to zero very quickly For this, the VMC approach can better avoid impacts, with a lower magnitude of the power peak, as a consequence of zeroing stiffness $k_\text {v}$ when the hip position is higher than $x_+$. Similar impact avoidance is observed in human hopping too.

The negative peak power results from the negative acceleration of the carriage driving the test bed and the high force acting on the cable, after reaching return point. As in this phase no force can be exerted on the leg, it does not affect its motion, but causes high forces in the drive train. Thus, smaller peaks, obtained by VMC is of advantage.

**Fig. 6:**
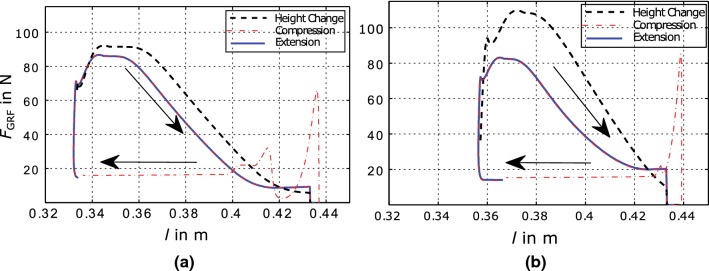
Work loops (relationship between leg length *l* and ground reaction force $F_\text {GRF}$) for simulations of MARCO-Hopper II with the ground level perturbation. **a** VMC approach with ${\varDelta }W=8\,{\hbox {J}}$, and ${\varDelta }W=10\,{\hbox {J}}$ for regular and increased hopping height, respectively, while $x_-=0.35\,{\hbox {m}}$ and $x_+=0.44\,{\hbox {m}}$. **b** Feed-forward control approach with $i_{\text {max}}=25{\hbox {A}}$ and $i_{\text {max}}=29{\hbox {A}}$ for regular and increased hopping height, respectively, and a frequency of $f_0=1.8\,{\hbox {Hz}}$ with a phase lag of $\phi _\text {c}=\pi /2$ (color figure online)

The consumed power in the acceleration phase is higher after the change of ground level for both approaches. This deviation of the power, the first peaks after the first red line in Fig. 4, is much higher for the feed-forward than for the VMC approach. As shown in Fig. 5, this results in a higher hopping height for the first hop after the perturbation. In Kalveram et al. (2012), an increase in hopping height happens after the disturbance of the ground level for human experiments. In general, human perturbation recovery is closer to results of the VMC approach than the feed-forward control. For reaching higher hopping height, the current and correspondingly the power increase. Again the consumed power for the feed-forward case is higher than for the VMC approach.

*The work-loop method* shows that the ground reaction force and the leg length show a linear relationship during human hopping motions (Maykranz et al. 2013). The corresponding proportionality factor can be interpreted as the effective stiffness of the human leg (Blum et al. 2009). In Fig. 6, the work loops for both control methods for one isolated hop are shown. Bold blue lines show leg extension, generated by the active phase of the actuator. Dash-dotted red line shows the leg shortening after touchdown when no actuation is exerted. The black dashed line shows one hop with increased hopping height.

Both approaches result in comparable maximum values of ground reaction forces, $F_\text {GRF}\approx 80\,{\hbox {N}}$. In feed-forward control, the range of motion $[0.36\,{\hbox {m}}, 0.44\,{\hbox {m}}]$ is smaller than in the VMC $[0.33\,{\hbox {m}}, 0.44\,{\hbox {m}}]$. One difference between the controllers performances is the level of ground reaction force near the fully extended configuration. With the VMC approach, the ground reaction force falls underneath the weight of the leg ($F_\text {m,tot}$), constant part of the red line at $F_\text {GRF}\approx 18\,{\hbox {N}}$. In the feed-forward approach, the force is always over this limit when the actuator accelerates the leg until hitting the knee stop, while acceleration in the VMC approach ends earlier. During the constant phase of the ground reaction force, the negative motion velocity is fixed.Considering the linear region of work loops, both methods result in leg stiffness values of about $k\approx 1350\,\hbox {Nm}^{-1}$. In Batts et al. (2016), Batts et al. found a similar stiffness ($1200\hbox {Nm}^{-1}$) for robot hopping with comparable masses, dimensions and negligible damping.

The *efficiency*$\eta _\text {h}$ per hop is indicated in Table 3. The efficiency for both approaches lies always in the same region. The feed-forward approach shows a more efficient result in the case of ground perturbation, while the VMC copes better with the changed hopping height. In the fourth row of the table, mean values of the efficiency during an undisturbed hopping motion are shown.

**Table 3:** Efficiency $$\eta _\text {h}$$

	VMC	Feed-forward
Ground perturbation	$\eta _\text {h}=0.3824$	$\eta _\text {h}=0.4975$
Hopping height change	$\eta _\text {h}=0.6250$	$\eta _\text {h}=0.5411$
Third hop after height change	$\eta _\text {h}=0.5918$	$\eta _\text {h}=0.6088$
Mean during continuous hopping	$\eta _\text {h}=0.7171$	$\eta _\text {h}=0.7017$

### MARCO-Hopper II in comparison with human hopping

**Fig. 7:**
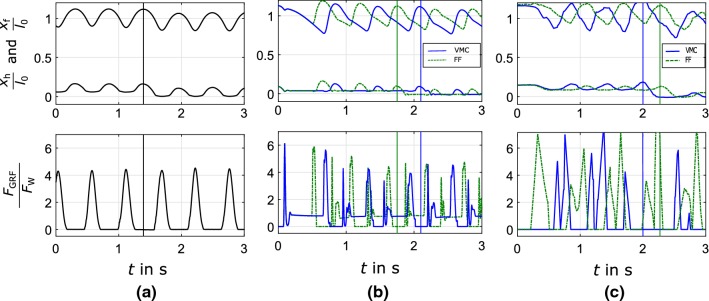
Hopping experiments with ground level perturbations (vertical lines) during flight phase, including the normalized GRF ($\frac{F_\text {GRF}}{F_\text {W}}$), hip $\frac{x_\text {h}}{l_\text {0}}$ and foot position ($\frac{x_\text {h}}{l_\text {0}}$). **a** Human hopping experiment adapted from Kalveram et al. (2012). The maximal leg length is $l_0=1.09\,{\hbox {m}}$, the weight is $F_\text {W}=800\,{\hbox {N}}$. **b** Simulation of MARCO-Hopper II. In VMC approach ${\varDelta }W=8\,{\hbox {J}}$. In the feed-forward approach $f_0=1.8\,{\hbox {Hz}}$, $i_\text {max}=25{\hbox {A}}$, $\phi _\text {c}=\pi /2$. The ground level changes by ${\varDelta }x=0.02\,{\hbox {m}}$, the maximal leg length is $l_0=0.44\,{\hbox {m}}$, the weight is $F_\text {W}=15\,{\hbox {N}}$. **c** Experiment with MARCO-Hopper II. In VMC approach ${\varDelta }W=8\,{\hbox {J}}$. In the feed-forward approach ($f_0=1.8\,{\hbox {Hz}}$, $i_\text {max}=17\hbox {A}$, $\phi _\text {c}=\pi /2$). The ground level changes similar to (**b**) (color figure online)

The performance of the two control approaches in comparison with human hopping is examined. Hopping experiments with ground level perturbations are conducted with human subjects, the simulation model and the MARCO-Hopper II hardware setup. The human experiments are described in Kalveram et al. (2012). In the new simulations, the change of ground level happens during the flight phase, as in the human experiments. We compare different characteristics including (i) hopping condition: frequency, duty factor, i.e., stance time to cycle time, (ii) kinematics: hip and foot position and knee angle, and (iii) kinetics: GRF. In Fig. 7, the solid and dashed lines illustrate the VMC and feed-forward approaches results, respectively. The occurrence of the perturbation is marked by vertical lines. A time window of four hops for each experiment is shown, to identify the perturbation effect.

All position values are relative to the maximal leg length, defined as the leg length just before the lift-off. For fair comparison, the GRFs are normalized to the corresponding body weights.

One difference between the human and robot hopping lies in the motion symmetry. The control approaches of MARCO-Hopper II include three phases: (1) acceleration; from maximum compression (MC) to takeoff (TO), (2) free motion; from TO to touchdown (TD) in which the cable is slack, and (3) deceleration; from TD to MC. Switching between phases 2 and 3 can be distinguished from the breaking point in the course of the hip position during downward motion (see Fig. 7b). This asymmetric pattern is also observable in the ground reaction force. The acceleration part of the movement (leg extension) is similar to the human hopping pattern starting with a large peak in GRF, followed by a valley with zero force, showing phase 2. Unlike the smooth increase of GRF in human hopping, in phase 3 of robot motion, a small peak followed by a plateau region and an increase are observed. Implementing control approaches on the hardware results in deviations from the simulation model while they are qualitatively similar, as shown in Fig. 7c.

A quantitative comparison between human and robot (simulation and experiments) hopping is presented in Table 4. The robot hopping frequency is close to 2 Hz, observed in human hopping experiment. The duty factor of robot hopping with VMC approach (45%) is the most similar one to human hopping (39%). By normalizing the hip (${\varDelta }l_\text {hip}$) and foot (${\varDelta }l_\text {hip}$) displacements to the corresponding leg length, the numbers from robot simulation and experiments are close to those of human experiments. In general, the hip motion in the robot is larger and the hopping height is smaller than in human hopping. Furthermore, the knee angle at touchdown ($\varphi _{k}^{TD}$) and maximum compression ($\varphi _{k}^{MC}$) are compared. It is shown that the closest knee angular displacement (${\varDelta }\varphi _{k}$) to human hopping ($21^\circ$) is achieved by implementing the VMC method on the robot ($23^\circ$). Finally, the normalized GRF in simulations are close to human hopping and these numbers are larger in robot experiments. Still, VMC method has a closer normalized GRF to human experiment. The results show that using the proposed template-based control approach the robot can mimic human hopping.

**Table 4:** Comparison between human and robot hopping. Mean values of different hops are shown

Measure	Human	Robot simulation		Robot Experiment	
		VMC	FF	VMC	FF
Frequency	2 Hz	1.6 Hz	1.8 Hz	1.8 Hz	1.7 Hz
Duty factor	39%	74%	63%	45%	67%
${\varDelta }l_\text {hip}$	0.25	0.35	0.3	0.32	0.27
${\varDelta }l_\text {hop}$	0.1	0.07	0.1	0.07	0.05
$\varphi _k^{TD}$	$136^\circ$	$123^\circ$	$123^\circ$	$105^\circ$	$110^\circ$
$\varphi _k^{MC}$	$115^\circ$	$86^\circ$	$94^\circ$	$82^\circ$	$96^\circ$
${\varDelta }\varphi _k$	$21^\circ$	$47^\circ$	$29^\circ$	$23^\circ$	$14^\circ$
$\frac{F_\text {GRF}}{F_\text {W}}$	4.5	4.4	4.8	6	6.9

## Discussion and conclusions

This paper compares two methods (feedback and feed-forward control) to achieve stable human-like hopping motions with a segmented leg in simulations and experiments on a robot. Both approaches are able to handle the desired hopping tasks while the following advantages of the VMC as the feedback control approach can be found:

1. Faster recovery from (ground level) perturbations than the feed-forward approach (Fig. 5)
2. Faster adaption to a new desired hopping height (Fig. 5)
3. Lower abrupt loads on the drive train during the movement (Fig. 4)
4. Freedom in selecting the initial condition (unlike feed-forward method, in which initial conditions depend on the phase lag $\phi _\text {c}$, Sect. 2.1.1)
5. Straightforward adaptation to variations, e.g., changing hopping height or changes in the environment, using sensory data
6. Replication of human hopping in the robot (Table 4).

These observations recommend using the VMC approach for the implementation of locomotion tasks. Changes in ground properties, gait types and conditions, e.g., hopping height, and system properties are handled better by the model-based method. Beyond the main focus of this study, we exploratively tested effects of ground compliance on the virtual leg stiffness in our simulations, which were in line with findings in human hopping reported in Ferris and Farley (1997).

The major drawback of the feed-forward control is a lacking straightforward method for finding the parameters. Experiments show that an adjustment of hopping frequency and current amplitude is only possible in a small parameter range to achieve stable hopping. In contrast, the bio-inspired VMC approach relies on mechanical properties that can be easily tuned and would even facilitate simple extension to other gaits. However, the main advantage of the feed-forward approach is that neither sensor nor biomechanical modeling is required to reach stable hopping with MARCO-Hopper II.

Mimicking human hopping is the next achievement of the proposed bio-inspired feedback control approach. Comparisons in Fig. 7 and Table 4 show similarities between human and robot experiments regarding hopping characteristics, kinematic and kinetic behavior. In that respect implementation of VMC on the robot could show the most similar results to human hopping. In this case, the differences between human and robot performance in hopping frequency, duty factor and knee angle displacement are 10–15%, while for the hip relative displacement, normalized hopping height and GRF remain below 30%. In this regard, the template-based model and its implementation on the robot can predict human behavior acceptably. The differences of the relative hopping height and hip motion are explainable through missing foot in two-segmented leg and the losses of the mechanism. Differences and fluctuations of the ground reaction force are due to the sensor setup. Yet, the elastic behavior of the human leg cannot be represented ideally with the actual setup. As shown in Sect. 3.2, the ground reaction force of the human indicates a storage of energy while decelerating the body. Also, work-loop diagrams of human hopping indicate that energy is stored during each hopping cycle (Maykranz et al. 2013), which is similar to findings in robot hopping. Although there is no elastic element in the drive train to store energy, the calculated virtual leg stiffness is close to the optimal value found in Batts et al. (2016).

Stable hopping motions with a segmented leg, using the VMC approach and the concept of constant energy supply, have been shown in the authors previous study (Oehlke et al. 2016). Here, the work is extended regarding robustness against perturbations, adaptation to reach new hopping heights and a comparison to human hopping. In addition, a feed-forward control which is loosely motivated by the CPG concept is implemented and compared to our bio-inspired VMC approach. In contrast to the feed-forward method which works without sensory feedback, the virtual model control emulating a leg spring works similarly to the reflex principle. The minimal feedback control of VMC mimics reflex control to create a desired actuator activity based on different biomechanical models (Blickhan 1989b) and constant energy supply (Kalveram et al. 2012), both working in an exploitive manner. The motion results from the combination of passive leg dynamics and active control.

Our template-based VMC method is able to mimic human hopping in a robot with a simple control architecture. Compared to a feed-forward technique, this feedback control shows better performance in terms of perturbation recovery and locomotion adaptation. The systematic tuning mechanism and bio-inspired concept of this approach may bring robots closer to the performance and versatility of their biological counterparts. For instance, it might help design prostheses that assist impaired humans in different gaits.

In the hierarchical sensorimotor neuromuscular control suggested by Loeb et al. (1999), preflexes are defined as the intrinsic properties of muscles at the lowest level of the hierarchy. These muscle properties lead to immediate responses to length and particularly velocity perturbations (Brown and Loeb 2000). This programmable high gain and immediate (zero delay) responsive property of muscle may be implementable in the lower level compared to the reflex control. Feed-forward control exhibits similarities to human preflex behavior, which enables efficient generation of repetitive motion while being limited in handling changes of the desired motion or influences of external perturbations. In contrast, VMC implements reflex-like structures and thereby facilitates reactions to changes. Relating to the concept of sensory-motor maps (Schumacher and Seyfarth 2017; Loeb et al. 1999), the present paper substantiates the suitability of using muscle fiber length and muscle force feedback as reflex pathways which is in line with findings in Haeufle et al. (2010).

The goal of this study was investigating the ability of the bio-inspired control method to mimic human hopping. Using the machine for human hopping prediction and also design of assistive devices are steps to be addressed in future of this research. An addition of compliance to the actuators (e.g., using serial elastic actuation) is a possible next step to improve efficiency and robustness against perturbations. Furthermore, the system could be extended to a three-segmented leg, including an active or passive foot. A controlled movement of this human-like structure can reveal further insights into the biomechanics of locomotion. For example, benefiting from zig-zag configuration of leg and biarticular actuation as two useful bio-inspired morphological design concepts can be targeted in the future. This way, the proposed approach could better predict human behavior.
